# GWAS and Selective Sweep Analysis Reveal the Genetic Basis of Papilla Number in the Sea Cucumber (*Apostichopus japonicus*)

**DOI:** 10.3390/ani16010066

**Published:** 2025-12-25

**Authors:** Yibo Wang, Jian Zhang, Zixin Hong, Fengqin Wang, Zhenping He, Miaomiao Yao, Hai Ren, Shanshan Yu, Qinglin Wang, Chunlong Zhao

**Affiliations:** 1 College of Marine Resources & Environment, Hebei Normal University of Science & Technology, Qinhuangdao 066000, China; yibowang5@163.com (Y.W.); zj7782133@163.com (J.Z.); eenoee@foxmail.com (Z.H.); wfq38161212@163.com (F.W.); hezhenping@163.com (Z.H.); 16608610141@163.com (M.Y.); renhai@163.com (H.R.); 2 Hebei Key Laboratory for Ocean Dynamics, Resources and Environments, Qinhuangdao 066600, China; 3 Ocean Fisheries Science Research Institute of Hebei Province, Qinhuangdao 066000, China; zhaochunlong@163.com

**Keywords:** GWAS, selective weep analysis, SNP, candidate gene

## Abstract

Sea cucumber (*Apostichopus japonicus*) is a commercially important mariculture species in northern China. Papilla number has been recognized as a key economic trait in sea cucumbers. Notably, significant variation in papilla count exists among different populations. The genetic mechanisms controlling papilla development are not fully understood. In this study, 72 individuals from six geographically distinct sea cucumber populations (Group N1) and 35 individuals from their offspring (Group N2) were analyzed using reduced-representation genome sequencing (RRGS) and whole-genome resequencing (WGS), respectively. Genome-wide association studies (GWAS) and selective sweep analysis were conducted to identify the biological pathways and genetic basis underlying variation in papilla number. The GWAS analysis identified two single-nucleotide polymorphism (SNP) loci on chromosomes 4 and 14 in the Group N1 that were significantly associated with papilla number. Within the vicinity of two SNPs, 48 genes were annotated as putative candidate genes, six of which have been reported to be associated with growth in *A. japonicus* or other aquatic animals. Selective sweep analysis identified 23 candidate genes in the JZ vs. YT within Group N1 and 39 candidate genes in the G1 vs. G3 within Group N2. Notably, functional enrichment analysis revealed that the Calcium signaling pathway was significantly enriched in both Group N1 and Group N2. This pathway has been demonstrated to regulate key cellular processes such as cell proliferation and differentiation through the activation of downstream signaling cascades. The intersection of results from parental Group N1 and progeny Group N2 yielded a total of six key biological pathways, including biological process, cellular process, cellular anatomical entity, cellular component, membrane, and binding. Collectively, our findings contribute to a deeper understanding of the genetic mechanisms underlying papilla number variation in *A. japonicus* and provide valuable insights for genomic selection in breeding programs.

## Simple Summary

The sea cucumber *Apostichopus japonicus* (*A. japonicus*) is a commercially valuable species due to its high nutritional and medicinal values. papilla number is one of the key economic traits in *A. japonicus*, closely associated with market grading standards in China. In the present study, reduced-representation genome sequencing (RRGS) and whole-genome resequencing (WGS) were conducted on *A. japonicus* collected from six geographically distinct populations in northern China (Group N1) and their offspring (Group N2), respectively. Subsequently, genome-wide association study (GWAS) and selective sweep analysis were integrated to identify candidate genes and biological pathways associated with papilla number in *A. japonicus*. GWAS identified two single-nucleotide polymorphism (SNP) loci and 48 candidate genes in Group N1. Selective sweep analysis identified 23 and 38 candidate genes in the parental group and offspring group, respectively. Functional enrichment analysis among these candidate genes highlighted six enriched biological pathways potentially involved in papilla number regulation in *A. japonicus*. These results provide a solid basis for revealing the papilla development mechanism of this species and open up new possibilities for developing papilla-rich breeding in sea cucumber.

## 1. Introduction

The sea cucumber, *Apostichopus japonicus*, belongs to the phylum Echinodermata, the class Holothuroidea, the order Aspidochirotida, and the family Stichopodidae. In China, it is widely distributed in the Yellow Sea and the Bohai Sea, inhabiting shallow coastal areas with abundant food supply, slow water flow, and the absence of freshwater input [1]. With rapidly rising living standards, market demand and consumption of *A. japonicus* have steadily increased over the past decade. To meet this growing demand, *A. japonicus* aquaculture has experienced rapid growth in China. According to the Chinese Fishery Statistical Yearbook (2025), the number of *A. japonicus* seedlings has reached 66.998 billion, and total production has increased to 326,172 tons [2]. The main aquaculture models for this species include bottom seeding enhancement, cofferdam culture, pond culture, industrialized aquaculture, and raft or cage cultivation, with four major production regions already established [3,4]. Among these, Hebei Province leads the country in both the breeding and production of large-sized sea cucumber seedlings through industrialized aquaculture. The primary production areas include Changli County in Qinhuangdao City, as well as Caofeidian District and Laoting County in Tangshan City. According to incomplete statistics, the aquaculture water volume is estimated to reach 4 million cubic meters, supplying nearly 80% of the large juvenile *A. japonicus* nationwide.

Body weight, growth rate, and papilla number are key economic traits and represent the central focus in genetic breeding research [5,6,7,8,9]. Among these, papilla number significantly influences the market value of large-sized *A. japonicus* seedlings, particularly within the industrialized aquaculture systems of Hebei Province, China. When the papilla are multiple and their root diameter is thick, a notable price premium exists compared to seedlings with thinner and fewer papilla. In some cases, individuals with inferior traits are not only difficult to sell but may even be discarded. Consequently, papilla number has become an important target trait in *A. japonicus* breeding programs. To date, Chinese researchers have successfully developed nine sea cucumber varieties using group selection methods. Among them, the highly spiked cultivars “Anyuan No.1” and “Anyuan No.2” were derived from the Russian–Chinese hybrid population “Shuiyuan No.1”. During the breeding process, the papilla number of the parent generation directly influences the corresponding trait in the offspring. Previous studies have revealed considerable variation in papilla counts across *A. japonicus* populations in Asia. Specifically, populations from Russia, Japan, and China exhibit average papilla counts of 66.25 ± 8.11, 53.64 ± 7.88, and 29.57 ± 3.31, respectively [5]. In addition, it has also been reported that wild sea cucumbers in China possess four–six rows of papilla [10], with papilla numbers across different geographic populations ranging from 36.84 ± 3.31 to 50.50 ± 9.03, despite the use of a standardized counting method as described by Chang et al. [5]. How can these discrepancies be explained? The underlying mechanisms governing papilla formation and developmental patterns in *A. japonicus* warrant thorough investigation.

Currently, research on economic traits primarily utilizes methodologies such as genome-wide association study (GWAS) and selective sweep analysis. These approaches provide essential technical tools for studies focusing on the breeding of improved aquatic animal varieties, stress resistance traits, and population genetic evolution. GWAS has successfully identified SNPs associated with economically important traits in different aquatic species, including the large yellow croaker (*Larimichthys crocea*) [11], Atlantic salmon (*Salmo salar*) [12], channel catfish (*Ictalurus punctatus*) [13], tiger pufferfish (*Takifugu rubripes*) [14], and obscure pufferfish (*Takifugu obscurus*) [15], among others. In addition, selective sweep analysis is currently widely applied in the identification of functional genes in aquatic animals. For instance, Wang et al. [16] employed selective sweep analysis to identify 76 and 99 candidate genes in two strains of Zhikong scallops. Sun et al. [17] sequenced 149 largemouth basses and detected genomic regions containing potential trait-associated genes, predicting 103 putative candidate genes under selection. Lin et al. [18] identified 349 positively selected genes, including *GHSR*, *HSF1*, *HABP2*, and *Dna J*, in the southern subspecies of *Argopecten irradians* through selective sweep analysis. In addition to conducting GWAS and selective sweep analysis, the GO and KEGG databases can be employed for in-depth functional annotation of the identified candidate genes and gene function enrichment analysis. These analyses provide valuable references for the identification of markers associated with growth and economic traits in aquatic species.

To date, studies on the molecular mechanisms underlying the formation and development of sea cucumber papilla remain limited, and the geographical distribution and sample sizes of the studied populations vary [7,8,9,19]. Furthermore, no previous study has integrated GWAS with selective sweep analysis to investigate these mechanisms. Therefore, we conducted GWAS and selective sweep analysis on nine populations of *A. japonicus* (six of which are natural populations from northern China, and three are descendant populations) to identify SNP loci and candidate genes associated with papilla number. Subsequently, the candidate genes identified using the two methods were subjected to integrative analysis and functional annotation. We anticipate that this work will provide a theoretical foundation for the breeding of superior *A. japonicus* varieties.

## 2. Materials and Methods

### 2.1. Sample Collection and DNA Extraction

The study involved two groups of 107 *A. japonicus* (N1 = 72; N2 = 35). Individuals in Group N1 were from wild groups at a depth of ten meters in different regions of northern China that were about to enter the breeding season [20]: CH (Changhai City, Liaoning Province, *n* = 12, average wet body weight: 232.34 ± 35.19 g), WF (Wafangdian City, Liaoning Province, *n* = 12, average wet body weight: 373.23 ± 99.68 g), JZ (Jinzhou City, Liaoning Province, *n* = 12, average wet body weight: 384.58 ± 109.44 g), TS (Tangshan City, Hebei Province, *n* = 12, average wet body weight: 191.13 ± 25.11 g), RZ (Rizhao City, Shandong Province, *n* = 12, average wet body weight: 170.80 ± 26.06 g), and YT (Yantai City, Shandong Province, *n* = 12, average wet body weight: 166.47 ± 19.23 g). A total of 35 individuals identified as Group N2 (average wet body weight: 5.73 ± 2.95 g) were selected from the offspring of Group N1 in mixed breeding protocols and classified into three groups based on papilla number (G1 ≥ 54 papilla, 53 ≥ G2 ≥ 46 papilla, G3 ≤ 45 papilla). To guarantee genetic continuity between Group N2 and Group N1, we chose individuals without obvious defects (such as body wall damage or abnormal development) at the same developmental stage from the offspring population of Group N1 as the source for Group N2. The method for counting the number of papilla is as follows: Visually examine the dorsal and ventral surfaces of the body wall and count all clearly visible protrusions with the naked eye. Each specimen should undergo two independent counts. The relative deviation between each count and the average of the two should not exceed ± 10%. If this criterion is not satisfied, additional counts should be performed until two readings meet this requirement.

Subsequently, the body walls from each group were collected and immediately preserved in liquid nitrogen prior to DNA extraction. The quality and quantity of genomic DNA obtained from individuals of Group N1 (parental population) and Group N2 (offspring population) were assessed.

### 2.2. Library Construction and RRGS, WGS of N1, N2

In Group N1, reduced-representation genome sequencing (RRGS) was performed (BioProject ID: PRJNA1363736). The SNP loci associated with papilla number (a quantitative trait) across large sample sizes were screened. The main steps are as follows: the extracted DNA was fragmented via ultrasound and underwent end repair. A single nucleotide was appended to the 3′ end to prevent self-ligation. Subsequently, sequencing adapters containing specific index sequences were ligated for immobilization on the Flow Cell. After two rounds of purification using magnetic beads to eliminate impurities, the library with an approximate insert size of 400 bp was selected by 2% agarose gel electrophoresis. Finally, the library was serially diluted, pooled, denatured into single strands with NaOH, and subjected to paired-end sequencing (PE150) on the Illumina HiSeq platform (Illumina, San Diego, CA, USA). For the RRGS data, fastp (v0.20.0) was employed to filter raw reads using the sliding window approach.

Group N2 was similarly processed, and whole-genome resequencing (WGS) was performed with the core objective of detecting more comprehensive genomic variations, such as SNPs in trait-associated regions, relying on full-genome coverage. According to the Illumina TruSeq protocol, DNA fragmentation was performed using the Covaris system with the TruSeq^TM^ Kit (Illumina, San Diego, CA, USA). Subsequently, the target fragments were purified and selected using magnetic beads. The fragments were subjected to end repair with EndRepairMix (Illumina, San Diego, CA, USA). A single “A” base was added to the 3′ end, followed by adapter ligation and magnetic bead purification. The DNA fragments with adapters at both ends were enriched through PCR to obtain the final library. After quantification using Picogreen (Illumina, San Diego, CA, USA) and quality assessment with the Agilent 2100 (Illumina, San Diego, CA, USA), the multiplexed libraries were normalized to 10 nM, pooled in equimolar ratios, serially diluted to a final concentration of 4–5 pM, and sequenced in PE150 mode on the Illumina NovaSeq platform (Illumina, San Diego, CA, USA).

### 2.3. Candidate Genes Identification

#### 2.3.1. Genome-Wide Association Study

The genome-wide association study (GWAS) of papilla number was performed via EMMAX’s mixed linear model [21,22], with the first three principal components (PCs) as covariates for population structure correction. The results were visualized using Manhattan and QQ plots. And *p*-values were corrected using the Bonferroni method. Significance threshold was calculated as −log_10_ (0.05/N), and suggestive threshold as −log_10_ (1/N) (N = total SNPs in GWAS). Candidate regions significantly associated with the trait were defined based on linkage disequilibrium (LD) analysis results. Specifically, a genomic interval of ±221,000 bp around each SNP exceeding the suggestive threshold line was selected as the candidate region, using the LD decay distance at which r^2^ decayed to 0.014.

#### 2.3.2. Selective Sweep Analysis

A selective sweep analysis was performed on Group N1 and Group N2 separately to identify candidate genes potentially associated with papilla number. Overlapping selected regions within each single population were merged, and genes located within or overlapping these merged regions—according to reference genome annotation—were considered as candidate selective genes.

### 2.4. Enrichment Analysis of Candidate Genes

TopGO was employed to perform Gene Ontology (GO) enrichment analysis. The *p*-values were calculated using the hypergeometric distribution test, with statistical significance defined as *p* < 0.05. GO terms showing significant enrichment were identified by comparing the gene set against the whole genome background, thereby revealing the major biological functions associated with these genes. In addition, Kyoto Encyclopedia of Genes and Genomes (KEGG) pathway enrichment analyses were performed to identify the functional characteristics of DEGs. The False discovery rate (FDR) was calculated to control false positives proportion.

## 3. Results

### 3.1. Phenotype Statistics

**Figure 1:**
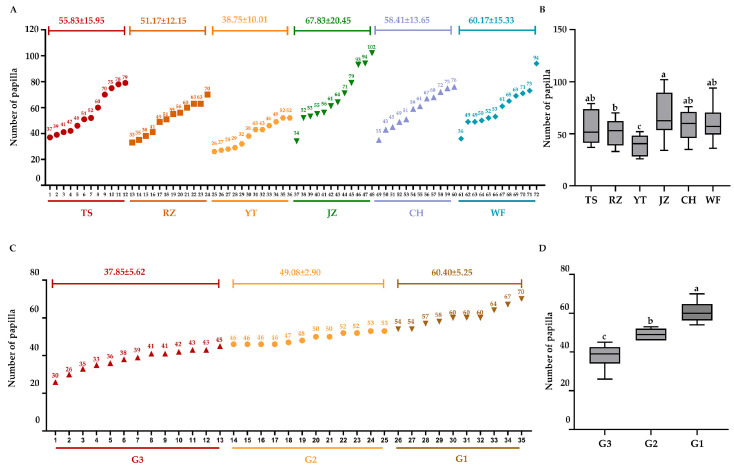
The number of papilla of *A. japonicus* in Group N1 and N2 and the significance analysis among them (each color in the figure corresponds to a population respectively). (**A**,**B**) Description about number and the significance analysis in Group N1. (**C**,**D**) Description about number and the significance analysis in Group N2. Different lowercase letters (e.g., a, b, c) were used to represent significant differences among groups; identical letters indicate no significant difference, whereas different letters indicate significant differences (*p* < 0.05).

We initially recorded the papilla counts from 72 *A. japonicus* individuals, representing six geographical populations within Group N1. As shown in Figure 1A, the JZ population exhibited the highest average number of papilla (67.83 ± 20.45), followed by WF (60.17 ± 15.33), CH (58.41 ± 13.65), TS (55.83 ± 15.95), and RZ (51.17 ± 12.15). In contrast, the YT population displayed the lowest average papilla count (38.75 ± 10.01). Combined with one-way ANOVA and Tukey HSD post-hoc tests, these results confirm a clear divergence in papilla number between the YT population and other geographical populations, especially the JZ population (Figure 1B).

In addition to Group N1, papilla number statistics and phenotypic observations were carried out for Group N2 (consisting of 35 offspring individuals). According to the classification criteria defined in Section 2, these 35 individuals were classified into three phenotypic groups with distinct papilla characteristics: G1: The papilla number ranged from 54 to 68. Papilla were densely distributed on both the dorsal and ventral body walls (as observed visually). G2: The papilla number ranged from 46 to 53, with a moderate papilla density and uniform distribution. G3: The papilla number ranged from 32 to 45. Compared to G1 and G2, the papilla distribution was sparse. Figure 1C,D visualizes the frequency distribution of papilla numbers and the significance analysis among them in Group N2. It clearly shows the non-overlapping median ranges and distinct phenotypic stratification among the three groups, providing a reliable phenotypic basis for subsequent selective sweep analysis.

### 3.2. SNP Genotyping of Group N1

**Figure 2:**
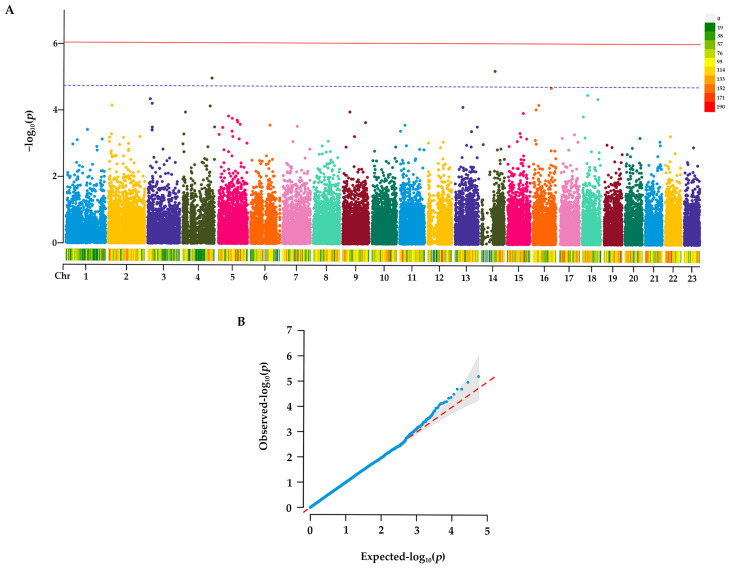
GWAS for genomic selection signals in *A. japonicus*. (**A**) Manhattan plots in the GWAS. Suggestive association cutoff of Manhattan plots (*p* < 1 × 10^5^) is denoted with a dashed line. Red solid line: Significance threshold line; blue dashed line: Indicative threshold line. (**B**) Quantile–quantile (QQ) plots in the GWAS. Blue dots in QQ plots represent the observed −log_10_(*p*) values for the study, and the red line denotes that the observed value is equal to the expected value.

Following quality control, approximately 99.88% of the raw reads were retained as high-quality clean reads, and a total of 55,016 SNPs were obtained in Group N1. GWAS was performed to detect SNPs associated with papilla count. Based on the EMMAX method analysis with Bonferroni correction, although no SNPs surpassed the genome-wide significance threshold, two SNPs reached the suggestive significance level. These SNPs were located at 33,752,760 bp on Chr4 and 15,241,244 bp on Chr14, respectively (Figure 2A).

Additionally, a QQ plot was generated to assess the reliability of the GWAS results. As shown in Figure 2B, the distribution of observed *p*-values in the initial segment of the plot is closely aligned with the expected null distribution represented by the reference line.

### 3.3. Candidate Genes Identification and Functional Annotation

#### 3.3.1. GWAS

**Figure 3:**
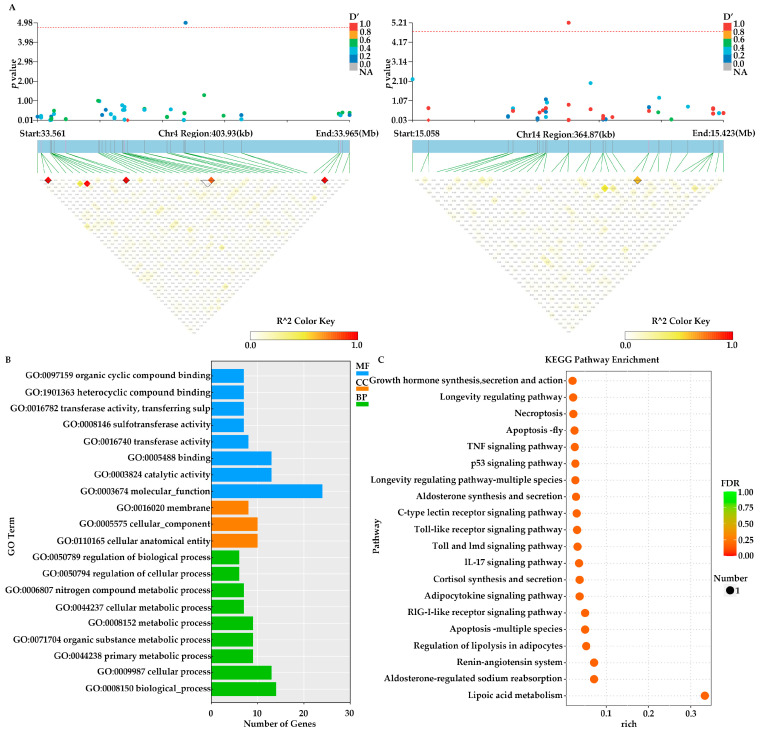
Functional annotations of GWAS (**A**) Local Manhattan plot of the candidate gene showing the significant SNP (top), the transcript in this region (middle) and the LD heatmap (bottom). (**B**) GO enrichment analysis of Group N1 was performed on the candidate genes identified through GWAS analysis. (**C**) KEGG enrichment analysis of Group N1 was performed on the candidate genes identified through GWAS analysis.

The candidate regions on Chr4 and Chr14 were scanned. After mapping these candidate regions to the *A. japonicus* genome (https://figshare.com/articles/dataset/The_genome_annotation_files_of_Apostichopus_japonicus/22140020/1?file=39361121, accessed on 27 December 2023), a total of 48 genes located within 221 kb upstream or downstream of the two SNP loci were annotated. Among them, six genes—*putative insulin receptor substrate 1*, *putative prolyl endopeptidase-like*, *putative ras-related protein*, *partial*, *putative phospholipase ABHD3*, *caspase-8*, and *putative FGFR1 oncogene partner 2-like*—have been reported to be associated with growth in *A. japonicus* or other aquatic animals (Figure 3A, Table 1).

To further elucidate the potential functions of the target genes, KEGG and Gene Ontology (GO) analyses were conducted on the 48 annotated genes. GO enrichment analysis identified the top 20 enriched terms. Of these, six were classified under Cellular Component (CC), eight under Molecular Function (MF), and six under Biological Process (BP). In the CC category, the most significantly enriched was “cellular anatomical entity (GO:0110165)” (10 genes), followed by “cellular_component (GO:0005575)” (10 genes) and “membrane (GO:0016020)” (8 genes). In the BP category, the most prominent term was “biological process (GO:0008150)” (14 genes), followed by “cellular process (GO:0009987)” (13 genes) and “metabolic process (GO:0008152)” (9 genes). For the MF category, the top term was “molecular function (GO:0003674)” (24 genes), followed by “catalytic activity (GO:0003824)” (13 genes) and “binding (GO:0005488)” (13 genes) (Figure 3B). Additionally, KEGG enrichment analysis revealed that the target genes were significantly enriched in several pathways, including “Growth hormone synthesis, secretion and action (ko04935)”, “Longevity regulating pathway (ko04211)”, “TNF signaling pathway (ko04668)” and “p53 signaling pathway (ko04115)” (Figure 3C).

#### 3.3.2. Selective Sweep Analysis

To identify candidate genes associated with papilla number, selective sweep analysis was conducted separately on individuals in Group N1 and Group N2. In Group N1, 23 candidate genes were identified in the JZ population relative to the YT population within putative selective regions (Table 2). Furthermore, in pairwise comparisons of CH, RZ, TS, and WF populations against YT, a total of 19, 19, 17, and 21 candidate genes were identified, respectively (Table S1). In Group N2, specifically in the comparison between G1 and G3, 39 candidate genes were detected within putative selective regions in the G1 population relative to the G3 population (Table 3). In the comparisons of G1 vs. G2 and G2 vs. G3, a total of 39 and 45 candidate genes were identified, respectively (Table S2).

**Figure 4:**
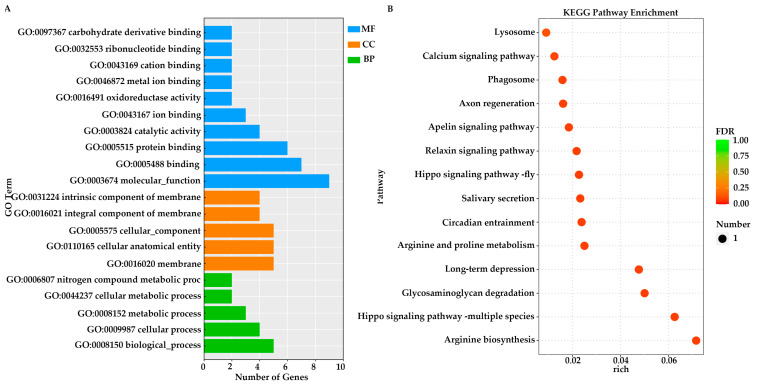

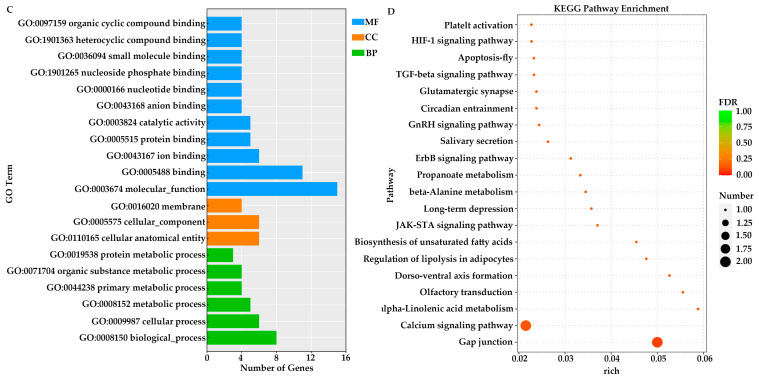
GO and KEGG enrichment analyses of two groups were performed on the candidate genes identified through selective sweep analysis. (**A**,**B**) JZ vs. YT; (**C**,**D**) G1 vs. G3.

GO and KEGG enrichment analyses were conducted on candidate genes identified from the two populations exhibiting the most significant differences in papilla count within Group N1 and Group N2, respectively. Specifically, in the comparison between the JZ and YT populations of Group N1, GO analysis revealed that the top three enriched terms were all associated with molecular functions: “molecular function (GO:0003674)”, “binding (GO:0005488)”, and “protein binding (GO:0005515)” (Figure 4A). In the G1 vs. G3 comparison, the first two enriched terms were identical to those observed in Group N1, followed by “biological processes” (GO:0008150) (Figure 4C).

In the KEGG enrichment analyses of these two groups, the molecular mechanisms underlying papilla number variation in *A. japonicus* were partially elucidated through the interaction among diverse pathways. In the JZ vs. YT population, KEGG enrichment was predominantly observed in signaling pathways—such as Steroid biosynthesis, Cholesterol metabolism and Regulation of lipolysis in adipocytes—that may significantly influence cell proliferation signals (Figure 4B). In the G1 vs. G3 comparison, enriched pathways included those related to ion transport (Calcium signaling pathway), cell proliferation, and growth factor (TGF-β signaling pathway, GnRH signaling pathway). Additionally, several metabolic pathways, including alpha-Linolenic acid metabolism and Propanoate metabolism, were also enriched (Figure 4D).

## 4. Discussion

In *A. japonicus* aquaculture, the number of papilla is considered a key economic trait, as it directly or indirectly reflects seedling rearing quality and potential economic return. According to our survey, spiny sea cucumber seedlings can be sold at a price of 100 CNY (1 USD = 7.12 CNY as of 9 November 2025) per kilo, which is 30–40 CNY higher than that of less spiny individuals at the wholesale price. In this study, significant differences in papilla count were observed among different geographical populations of sea cucumbers, which is consistent with the findings of previous studies [8,9]. Although the spine counts recorded in this study were higher than those previously reported, this discrepancy is primarily attributable to differences in counting methodology. In the present study, a more direct and intuitive approach was employed for papilla enumeration to reduce the likelihood of overlooking shorter papilla.

The nine currently recognized sea cucumber varieties in China have all been developed through traditional selective breeding methods. This approach entails a prolonged breeding cycle and requires multiple generations to achieve and stabilize the desired phenotypic traits. Therefore, modern breeding technologies are urgently needed to accelerate the genetic improvement of *A. japonicus* [20]. With advances in genetic improvement technologies, genes conferring production advantages are progressively enriched and fixed during selection, thereby generating detectable selection signals [23,24]. The identification of these selectively targeted genomic regions in genetically improved individuals facilitates the elucidation of the genetic basis underlying economically important traits [25,26]. Recently, transcriptome comparison, GWAS, and selective sweep analyses have been widely applied to the study of sea cucumber traits, including skin yield, polysaccharide, collagen, and saponin content, as well as growth and body weight [7,27,28,29,30]. Previous research had indicated that the phenotypic variations among different geographical groups are attributable to distinct instances of natural selection in diverse geographical locations [31,32]. Regarding the papilla number, significant differences have been observed among sea cucumber populations across different geographic regions. Although several studies have investigated papilla development [7,8,9,19], research on the molecular mechanisms underlying papilla formation and number variation has yet to produce definitive results. In this study, several significant features were used to systematically elucidate the molecular mechanisms underlying papilla number in sea cucumbers. First, this investigation is the first comprehensive integration of GWAS to employ selective sweep analysis to elucidate the genetic basis of papilla number in *A. japonicus*. Second, the study incorporated six geographically distinct wild populations collected from northern China, and their offspring, generated through mixed breeding protocols that closely mirror contemporary aquaculture practices, were also included in the analysis. Third, a novel, high-quality chromosome-level genome assembly of *A. japonicus* was utilized, providing improved genomic resolution for accurate variant detection and annotation [33].

Through a comprehensive analysis of the genome-wide association study (GWAS) results for Group N1 and the selective sweep analyses for Groups N1 and N2, multiple overlapping genetic elements and biological pathways were identified. In the GO enrichment analysis, terms such as biological process (GO:0008150), cellular process (GO:0009987), cellular anatomical entity (GO:0110165), cellular component (GO:0005575), membrane (GO:0016020), and binding (GO:0005488) were significantly enriched across all three analytical datasets. Among these, terms such as cellular process (GO:0009987), cellular anatomical entities (GO:0110165), and membrane (GO:0016020) have been widely reported to be enriched in growth-related studies [34]. Previous investigations into sea cucumber papilla development have demonstrated that growth-associated pathways play a crucial role in papilla morphogenesis [7,8,9,19]. Given these findings, we propose that these GO terms may, similarly, play a significant role in the formation and growth of sea cucumber papilla. Notably, in the KEGG enrichment analysis, the Calcium signaling pathway was enriched in both the parental Group N1 and the progeny Group N2 based on selective sweep analysis. Ca^2+^ is a ubiquitous intracellular messenger that exists within cells either in free form or bound to calcium-regulated proteins. They play a crucial role in regulating a series of cellular physiological functions, ranging from growth and differentiation to apoptosis [35,36]. Additionally, previous research has demonstrated that Ca^2+^ can regulate the Hippo signaling pathway associated with apoptosis through its mediating effect [37]. Cui et al. found that the Hippo signaling pathway was enriched in their research on sea cucumber growth [29]. Based on our findings, we hypothesize that the Calcium signaling pathway is closely associated with the formation of papilla. In the future, more systematic investigations into the role of this pathway in determining the number of papilla in sea cucumbers are warranted.

The GWAS results for Group N1 were analyzed independently to investigate the genomic variations associated with papilla number. Two significantly associated SNP loci on Chromosome 4 (Chr4) and Chromosome 14 (Chr14) were identified, and a total of 48 genes were identified in genomic regions proximal to these SNP loci. Among these, six genes have been implicated in animal growth-related processes, including *Irs1* [38], *Prepl* [39], *Abhd3* [40], *Ran* [6], *Casp8* [41], and *Fgfr1op2-like* [40]. *Irs1* and *Prepl* play a crucial role in normal growth. In mammals, the absence of *Irs1* or *Prepl* results in severe growth impairment [38]. *Ran* is a member of the Ras superfamily of small GTPases, serving as a critical regulator in cell fate determination. Transcriptome analysis of sea cucumber papilla revealed that *Ran* plays a crucial role in regulating growth and morphogenesis [6]. Furthermore, *Fgfr1op2-like* has been demonstrated to be associated with cell growth and proliferation [40]. *Abhd3* is involved in the expression of proteins related to substance transport and metabolism [39,40]. In contrast, *Casp8* is highly significant in apoptosis regulation [41]. Zhu et al. previously demonstrated that proteins associated with cell cycle regulation, cell division, development, apoptosis, and growth may serve as key regulators in the formation and development of sea cucumber papilla, with alterations in cellular activities directly influencing papilla-related phenotypic traits [8]. By integrating these findings with the sea cucumber papilla transcriptome data reported by Zhou et al. [6], we propose that the aforementioned growth-related genes may play a significant role in the number variation of sea cucumber papilla.

In addition, through GO and KEGG enrichment analyses, several pathways and terms associated with growth traits were also enriched. For instance, in KEGG enrichment analysis, the “Growth hormone synthesis, secretion, and action” pathways were significantly enriched. Previous studies indicate that growth hormone (GH) secretion is regulated by the hypothalamic-pituitary axis, primarily through an antagonistic balance between growth hormone-releasing hormone (GHRH) and somatostatin (SST) [42]. Among them, GnRH and GHRH jointly act on the hypothalamic-pituitary axis in the subsequent signal transduction process [43]. Notably, Zhu et al. previously reported enrichment of the GnRH signaling pathway in relation to papilla number in sea cucumbers [8]. We assume that growth hormone may facilitate cell proliferation and differentiation, thereby influencing papilla number. In the GO enrichment analysis, the term “Apoptosis-multiple species” was significantly enriched (Figures S1–S6). This implies that papilla development in *A. japonicus* may depend on the dynamic interplay between cell proliferation and apoptosis. The p53 signaling pathway, which can be affected by viral infections and environmental stresses, may influence the expression of apoptosis-related genes [44,45]. During the formation and development of papilla, apoptosis may govern both morphology and number.

In addition, the TNF and IL-7 signaling pathways serve as a “regulatory hub” integrating immunity and metabolism. Chen et al. [46] demonstrated that the TNF family participates in inflammatory responses and immune defenses, thus modulating inflammation and apoptosis. Zhang et al. [47], studying grass carp, showed that TNFα may induce growth retardation under certain conditions during aquaculture. As for the IL-7 signaling pathway, Cui et al. [29] also enriched this pathway in their study of SNPs in the context of the growth of sea cucumbers. These findings suggest that the formation of papilla in *A. japonicus* may also be related to the immune microenvironment. Therefore, we hypothesize that certain enriched pathways—particularly the Growth hormone synthesis, secretion, and action pathway—may be functionally linked to variation in papilla number.

## 5. Conclusions

In summary, we integrated GWAS and selective sweep analysis to elucidate the genetic basis of the papilla number trait. First, we investigated papilla number in sea cucumbers from six geographically distinct populations (Group N1). Through GWAS, two SNP loci associated with papilla number were identified on chromosomes 4 and 14, respectively, along with 48 candidate genes. Among them, six genes, including *Irs1*, *Prepl*, *Ran*, *Abhd3*, *Casp8*, and *Fgfr1op2-like*, have been confirmed to be closely associated with the growth of aquatic animals. Subsequently, the parental Group N1 and offspring groups N2 were analyzed using selective sweep analysis. In the JZ vs. YT and G1 vs. G3 comparisons, 23 and 39 candidate genes were identified, respectively, which are enriched in biological processes such as ion binding, protein binding, and the calcium signaling pathway. By integrating GWAS and selective sweep analysis, six biological pathways associated with sea cucumber papilla growth—biological process, cellular process, cellular anatomical entity, cellular component, membrane, and binding—were consistently enriched in both Group N1 and Group N2. This study can provide a theoretical basis for analyzing the genetic structure of papilla number in *A. japonicus* and provide data support for upcoming molecular mechanism research and genome editing-assisted breeding.

## Figures and Tables

**Table 1:** Candidate genes associated with the papilla number traits based on GWAS in Group N1.

Gene ID	Gene Name	Gene ID	Gene Name
***evm.TU.Chr4.1192***	***putative insulin receptor substrate 1***	*evm.TU.Chr4.1217*	*putative galactose-3-O-sulfotransferase 4-like*
***evm.TU.Chr4.1193***	***putative prolyl endopeptidase-like***	*evm.TU.Chr4.1218*	
***evm.TU.Chr4.1194***	***putative ras-related protein, partial***	*evm.TU.Chr4.1219*	*hypothetical protein BSL78_05837*
*evm.TU.Chr4.1195*	*hypothetical protein BSL78_30215, partial*	*evm.TU.Chr4.1220*	*hypothetical protein BSL78_05837*
*evm.TU.Chr4.1196*		*evm.TU.Chr4.1221*	*hypothetical protein BSL78_23158, partial*
*evm.TU.Chr4.1197*		*evm.TU.Chr4.1222*	*putative solute carrier organic anion transporter family member 4A1, partial*
*evm.TU.Chr4.1198*		*evm.TU.Chr14.172*	*putative 5′-nucleotidase-like*
*evm.TU.Chr4.1199*	*putative F-box only protein 6 isoform X2*	*evm.TU.Chr14.173*	
*evm.TU.Chr4.1200*	*hypothetical protein BSL78_28705*	***evm.TU.Chr14.174***	***putative FGFR1 oncogene partner 2-like***
*evm.TU.Chr4.1201*		*evm.TU.Chr14.175*	
***evm.TU.Chr4.1202***	***putative phospholipase ABHD3***	*evm.TU.Chr14.176*	*putative homeobox protein DBX1-like*
***evm.TU.Chr4.1203***	***caspase-8***	*evm.TU.Chr14.177*	
*evm.TU.Chr4.1204*	*putative vascular endothelial growth factor receptor kdr-like*	*evm.TU.Chr14.178*	*hypothetical protein BSL78_24596, partial*
*evm.TU.Chr4.1205*	*putative heparan sulfate glucosamine 3-O-sulfotransferase 1-like*	*evm.TU.Chr14.179*	*hypothetical protein BSL78_25904*
*evm.TU.Chr4.1206*		*evm.TU.Chr14.180*	*hypothetical protein BSL78_25905*
*evm.TU.Chr4.1207*	*Serrate RNA effector molecule-like protein*	*evm.TU.Chr14.181*	*putative choline kinase alpha*
*evm.TU.Chr4.1208*		*evm.TU.Chr14.182*	*putative WSC domain-containing protein 2*
*evm.TU.Chr4.1209*		*evm.TU.Chr14.183*	*putative WSC domain-containing protein 1-like*
*evm.TU.Chr4.1210*	*hypothetical protein BSL78_28713*	*evm.TU.Chr14.184*	*putative WSC domain-containing protein 1-like*
*evm.TU.Chr4.1211*		*evm.TU.Chr14.185*	*putative WSC domain-containing protein 1-like*
*evm.TU.Chr4.1212*	*hypothetical protein BSL78_19697*	*evm.TU.Chr14.186*	*mediator of RNA polymerase II transcription subunit 18-like*
*evm.TU.Chr4.1213*	*hypothetical protein BSL78_28713*	*evm.TU.Chr14.187*	
*evm.TU.Chr4.1214*	*putative solute carrier family 35 member G1*	*evm.TU.Chr14.188*	*hypothetical protein BSL78_21844*
*evm.TU.Chr4.1215*	*hypothetical protein BSL78_05837*	*evm.TU.Chr4.1217*	*putative galactose-3-O-sulfotransferase 4-like*
*evm.TU.Chr4.1216*	*hypothetical protein BSL78_05837*	*evm.TU.Chr4.1218*	

The blank areas represent proteins that had not been annotated previously, and the bold font indicates genes that have been previously annotated as being associated with growth.

**Table 2:** Candidate genes associated with papilla number traits based on selective sweep analysis in JZ vs. YT populations.

Gene ID	Gene Name	Gene ID	Gene Name
*evm.TU.Chr9.624*	*N-acetylgalactosamine-6-sulfatase isoform X1*	*evm.TU.Chr2.1061*	*putative nesprin-1-like*
*evm.TU.Chr1.258*	*putative integrator complex subunit 12*	*evm.TU.Chr20.134*	*putative C-mannosyltransferase DPY19L1 isoform X2*
*evm.TU.Chr1.259*	*putative bifunctional arginine demethylase and lysyl-hydroxylase JMJD6-B-like*	*evm.TU.Chr22.439*	*putative ras association domain-containing protein 2 isoform X2*
***evm.TU.Chr11.278***	***NOS***	*evm.TU.Chr22.440*	*putative sperm-tail PG-rich repeat-containing protein 2*
*evm.TU.Chr13.548*	*putative E3 ubiquitin-protein ligase MYCBP2*	*evm.TU.Chr4.376*	*putative tyrosine kinase receptor Cad96Ca*
*evm.TU.Chr14.525*	*hypothetical protein BSL78_14137*	*evm.TU.Chr4.377*	
*evm.TU.Chr14.526*	*hypothetical protein BSL78_14138*	*evm.TU.Chr5.444*	
*evm.TU.Chr15.764*	*hypothetical protein BSL78_02677, partial*	*evm.TU.Chr7.539*	*hypothetical protein BSL78_02175*
*evm.TU.Chr15.765*	*cilia- and flagella-associated protein 157-like*	*evm.TU.Chr7.540*	
*evm.TU.Chr16.682*	*hypothetical protein BSL78_13135*	*evm.TU.Chr7.541*	
*evm.TU.Chr17.138*	*Nucleoplasmin-like protein ANO39, partial*	*evm.TU.Chr8.535*	*putative fibronectin-like*
*evm.TU.Chr2.1060*	*putative nesprin-1-like*		

The blank areas represent proteins that had not been annotated previously, and the bold font indicates genes that have been previously annotated as being associated with growth.

**Table 3:** Candidate genes associated with the papilla number traits based on selective sweep analysis in G1 vs. G3 populations.

Gene ID	Gene Name	Gene ID	Gene Name
*evm.TU.Chr9.1058*	*hypothetical protein BSL78_12224*	*evm.TU.Chr20.582*	*sorcin*
*evm.TU.Chr6.813*	*hypothetical protein BSL78_12013*	*evm.TU.Chr2.1387*	*putative DDB1- and CUL4-associated factor 5*
*evm.TU.Chr5.1112*	*dynein regulatory complex subunit 6-like isoform X2*	*evm.TU.Chr2.1386*	*hypothetical protein BSL78_04562*
*evm.TU.Chr4.1347*		*evm.TU.Chr19.688*	*trypsin-like serine protease*
*evm.TU.Chr4.1345*	*putative peroxisomal acyl-coenzyme A oxidase 1 isoform X3*	*evm.TU.Chr17.756*	*corazonin-like precursor*
*evm.TU.Chr4.1344*	*putative bystin-like*	*evm.TU.Chr17.395*	*putative non-structural maintenance of chromosomes element 4-like A-like*
*evm.TU.Chr23.470*	*epidermal growth factor receptor*	*evm.TU.Chr16.863*	*putative follistatin isoform X2*
*evm.TU.Chr23.469*		*evm.TU.Chr16.849*	*putative ADAMTS-like protein 5*
*evm.TU.Chr23.468*	*putative macrophage mannose receptor 1-like isoform X2*	*evm.TU.Chr14.570*	*vesicular glutamate transporter 1-like isoform X2*
*evm.TU.Chr22.620*	*hypothetical protein BSL78_12517*	*evm.TU.Chr13.726*	*putative acyl-CoA synthetase family member 2, mitochondrial-like*
*evm.TU.Chr22.619*		*evm.TU.Chr12.807*	
*evm.TU.Chr22.618*	*hypothetical protein BSL78_12516, partial*	*evm.TU.Chr12.806*	*hypothetical protein BSL78_23366*
*evm.TU.Chr22.617*	*cGMP-dependent protein kinase 1-like isoform X2*	*evm.TU.Chr12.805*	
*evm.TU.Chr21.711*		*evm.TU.Chr12.803*	*hypothetical protein BSL78_18289*
*evm.TU.Chr21.710*	*hypothetical protein BSL78_26158*	*evm.TU.Chr11.981*	
*evm.TU.Chr21.708*	*hypothetical protein BSL78_26159*	*evm.TU.Chr10.850*	
*evm.TU.Chr21.544*	*hypothetical protein BSL78_25588*	*evm.TU.Chr1.1191*	*putative UDP-D-xylose:L-fucose alpha-1,3-D-xylosyltransferase 3-like*
*evm.TU.Chr20.583*	*hypothetical protein BSL78_07486, partial*	*evm.TU.Chr1.1101*	*putative UDP-D-xylose:L-fucose alpha-1,3-D-xylosyltransferase 7-like*

The blank areas represent proteins that had not been annotated previously.

## Data Availability

The original contributions presented in this study are included in the article and Supplementary Material. Further inquiries can be directed to the corresponding authors.

## Supplementary Materials

The following supporting information can be downloaded at https://www.mdpi.com/article/10.3390/ani16010066/s1. Figure S1. Enrichment analysis of CH vs. YT populations was performed on the candidate genes identified through selective sweep analysis; Figure S2. Enrichment analysis of RZ vs. YT populations was performed on the candidate genes identified through selective sweep analysis; Figure S3. Enrichment analysis of TS vs. YT populations was performed on the candidate genes identified through selective sweep analysis; Figure S4. Enrichment analysis of WF vs. YT populations was performed on the candidate genes identified through selective sweep analysis; Figure S5. Enrichment analysis of G1 vs. G2 populations was performed on the candidate genes identified through selective sweep analysis; Figure S6. Enrichment analysis of G2 vs. G3 populations was performed on the candidate genes identified through selective sweep analysis; Table S1. Candidate genes associated with the papilla number traits in the CH, RZ, TS, WF vs. YT populations, respectively; Table S2. Candidate genes associated with the papilla number traits in the G1 vs. G2 and G2 vs. G3 populations, respectively.
