# Supplementary material for: GWAS and Selective Sweep Analysis Reveal the Genetic Basis of Papilla Number in the Sea Cucumber (Apostichopus japonicus)
Source: Animals (Basel). 2025 Dec 25;16(1):66. doi: 10.3390/ani16010066 (PMC12784826; doi:10.3390/ani16010066)
Supplement: Supplementary file 1 [file animals-16-00066-s001.zip › animals-3938259-supplementary.pdf]

Supplement materials

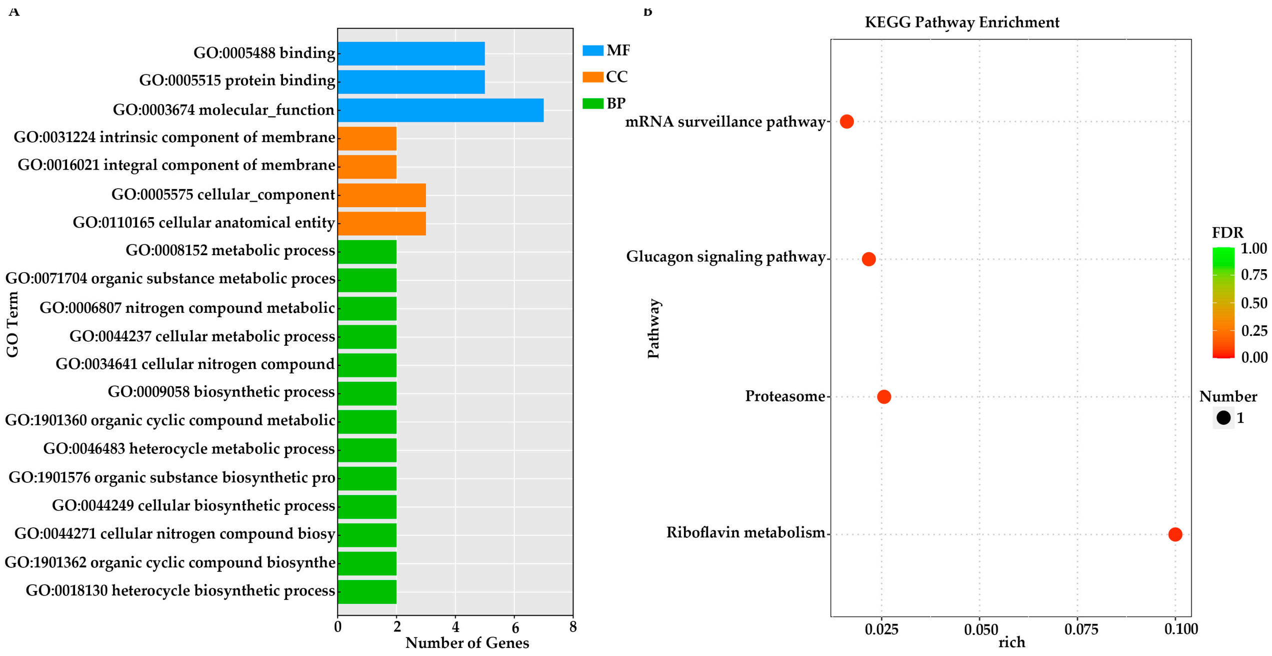

Figure S1. Enrichment analysis of CH vs. YT population was performed on the candidate genes identified through selective sweep analysis. (A) GO enrichment analysis (B) KEGG enrichment analysis.

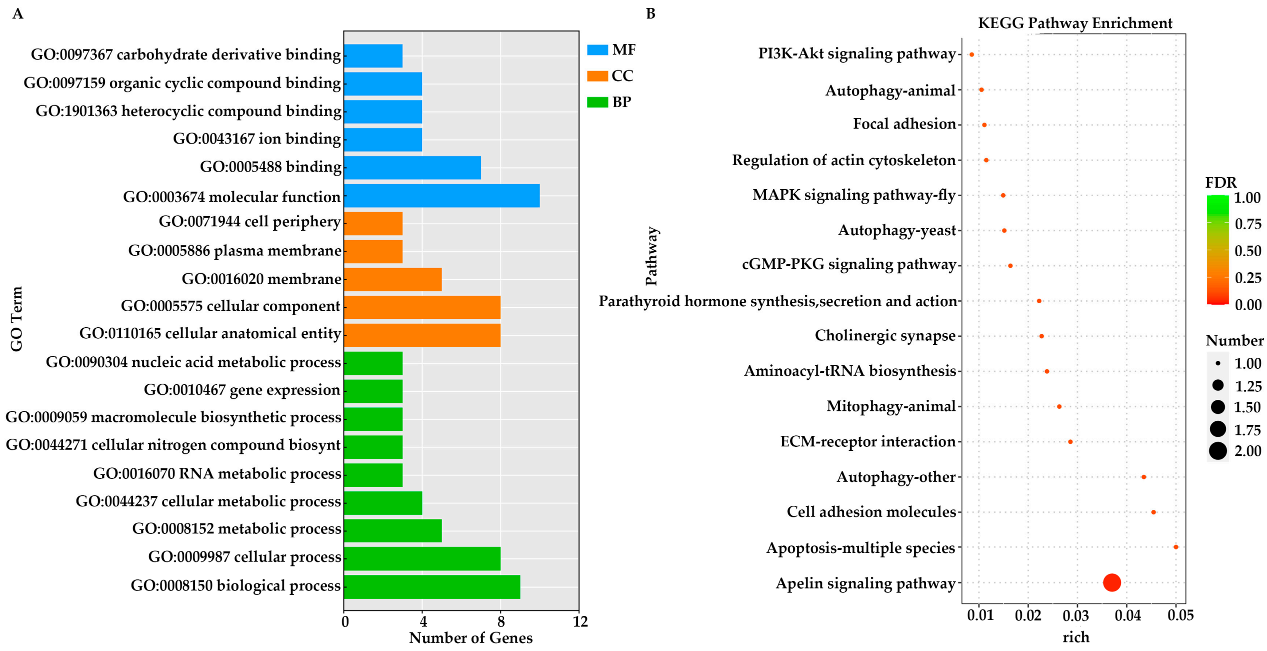

Figure S2. Enrichment analysis of RZ vs. YT population was performed on the candidate genes identified through selective sweep analysis. (A) GO enrichment analysis (B) KEGG enrichment analysis.

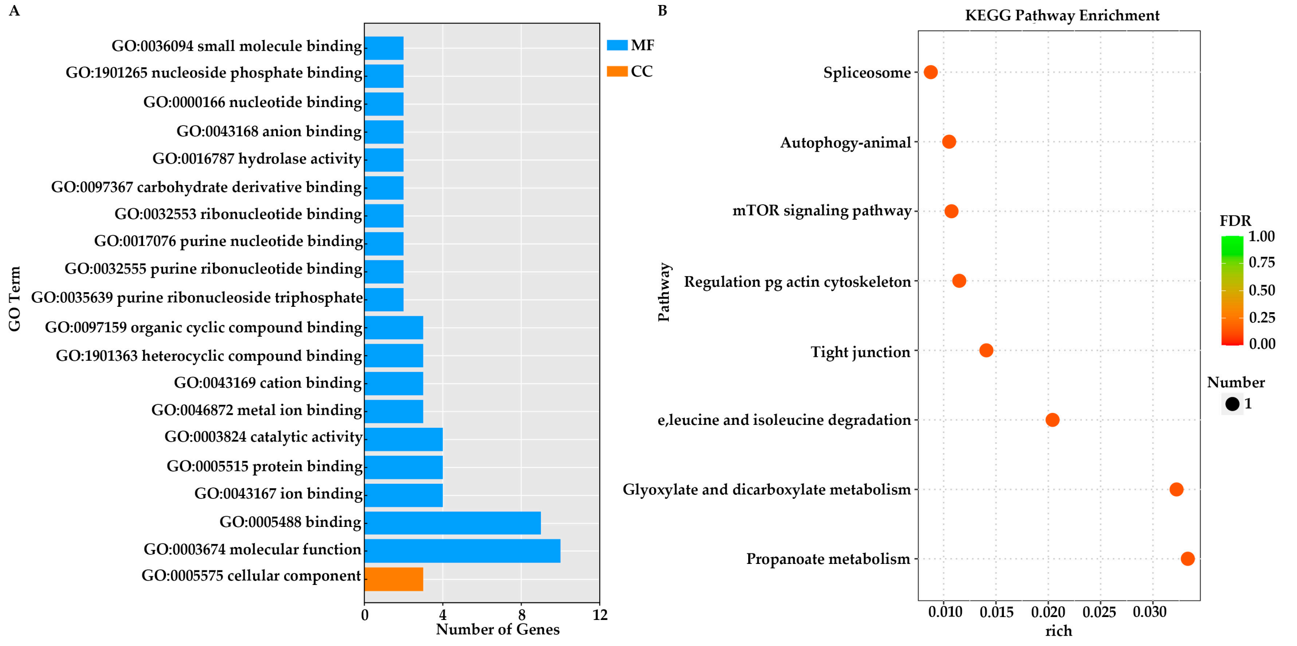

Figure S3. Enrichment analysis of TS vs. YT population was performed on the candidate genes identified through selective sweep analysis. (A) GO enrichment analysis (B) KEGG enrichment analysis.

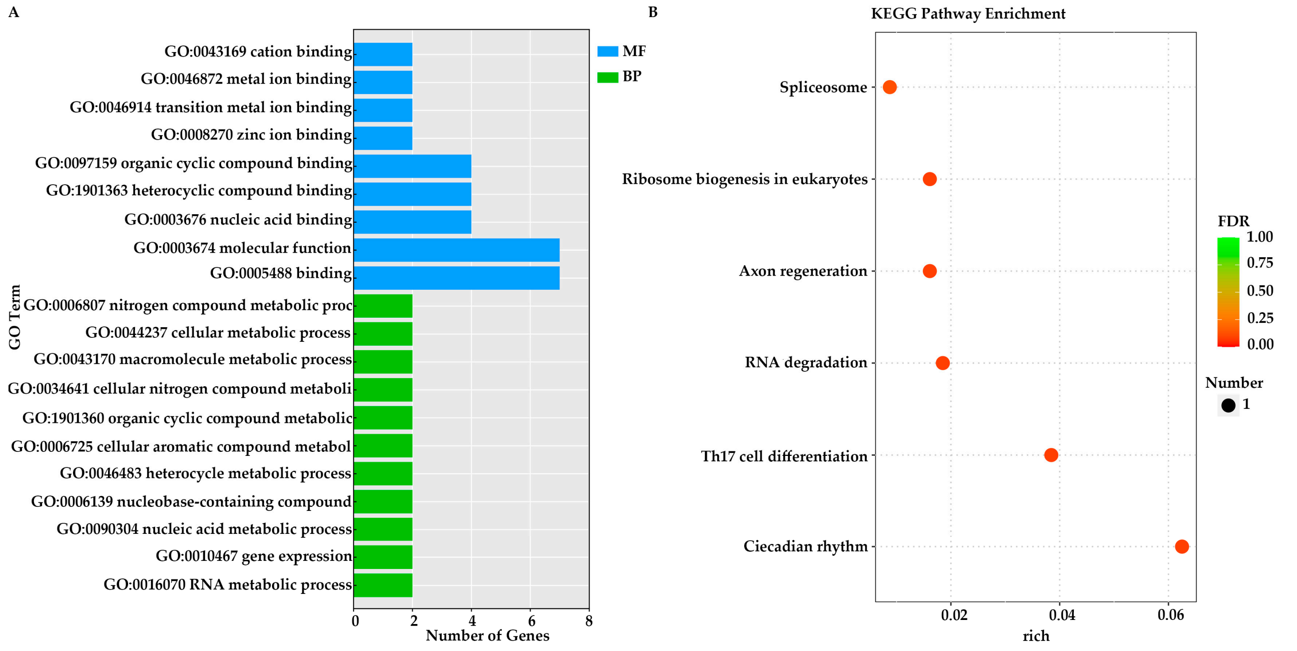

Figure S4. Enrichment analysis of WF vs. YT population was performed on the candidate genes identified through selective sweep analysis. (A) GO enrichment analysis (B) KEGG enrichment analysis.

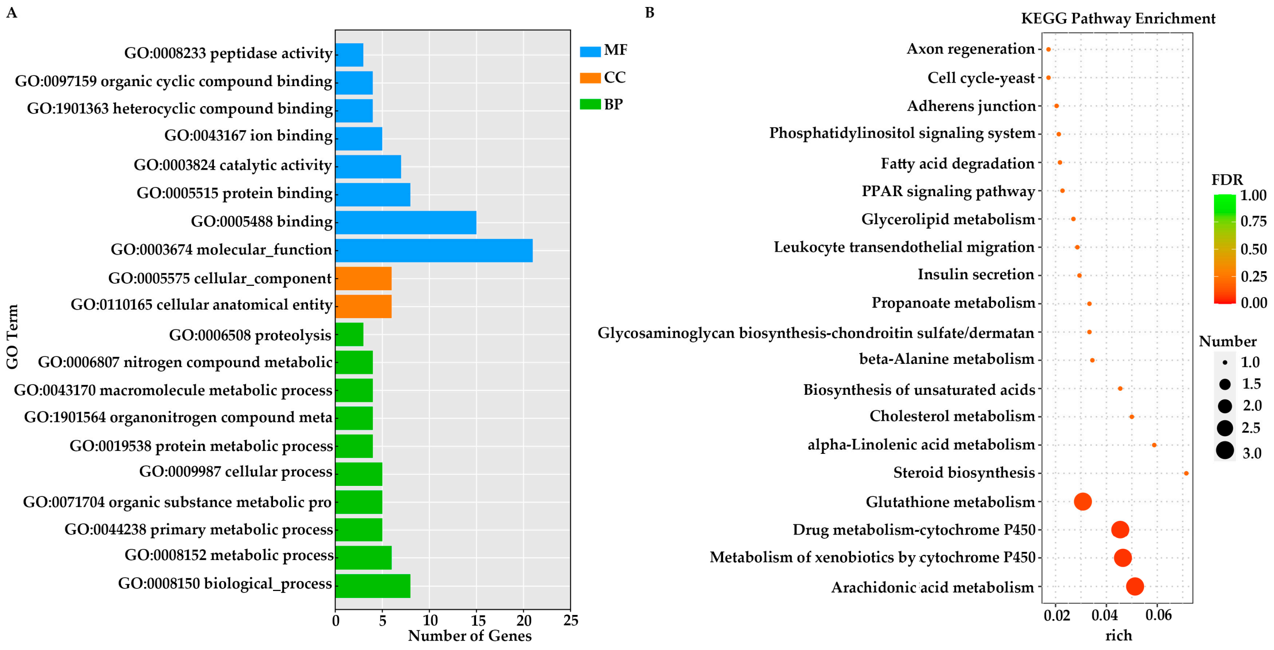

Figure S5. Enrichment analysis of G1 vs. G2 population was performed on the candidate genes identified through selective sweep analysis. (A) GO enrichment analysis (B) KEGG enrichment analysis.

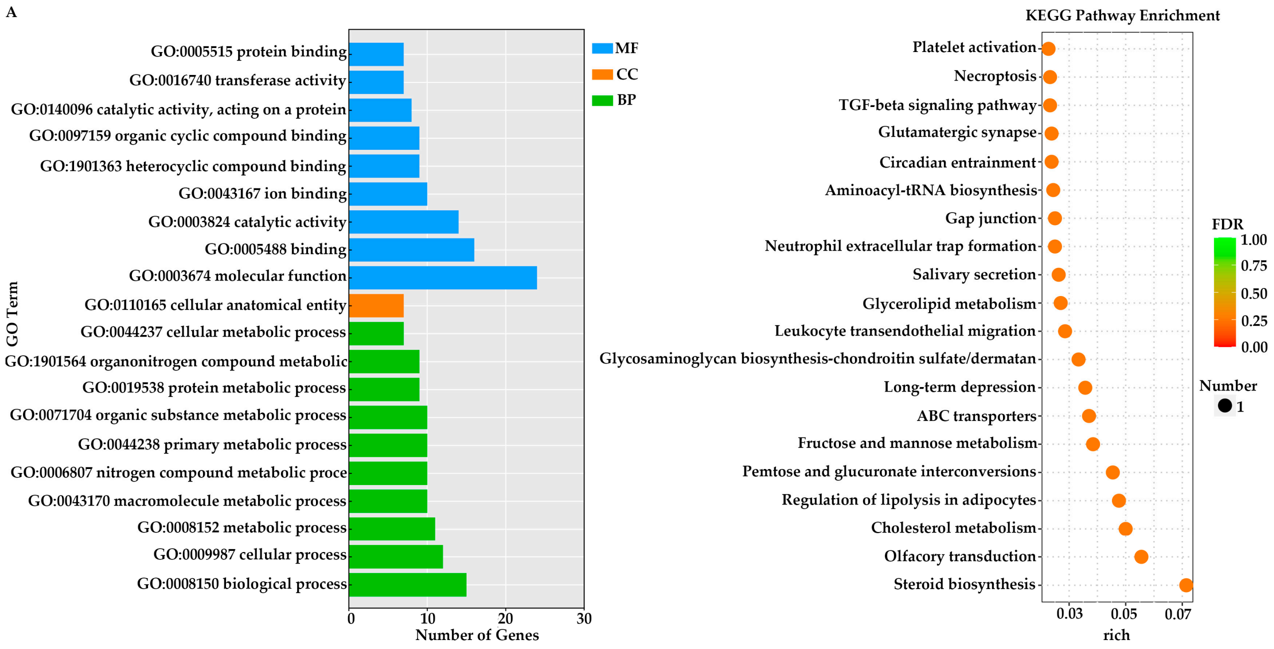

Figure S6. Enrichment analysis of G2 vs. G3 population was performed on the candidate genes identified through selective sweep analysis. (A) GO enrichment analysis (B) KEGG enrichment analysis.

Table S1. Candidate genes associated with the papilla number traits in the CH, RZ, TS, WF vs. YT populations, respectively.

CH vs. YT population		RZ vs. YT population		TS vs. YT population		WF vs. YT population	
**Gene ID**	**Gene Name**	**Gene ID**	**Gene Name**	**Gene ID**	**Gene Name**	**Gene ID**	**Gene Name**
evm.TU.Chr7.541		*evm.TU.Chr8.116*	*hypothetical protein BSL78_05512, partial*	*evm.TU.Chr9.662*	*hypothetical protein BSL78_05680*	*evm.TU.Chr9.483*	*putative NFX1-type zinc finger-containing protein 1-like*
evm.TU.Chr7.540		*evm.TU.Chr7.394*		*evm.TU.Chr7.481*	*hypothetical protein BSL78_03481, partial*	*evm.TU.Chr9.482*	*putative NFX1-type zinc finger-containing protein 1-like*
evm.TU.Chr3.367	*hypothetical protein BSL78_20126*	*evm.TU.Chr7.393*	*putative scavenger receptor cysteine-rich protein type 12 isoform X1*	*evm.TU.Chr6.651*		*evm.TU.Chr9.481*	*putative NFX1-type zinc finger-containing protein 1-like*
evm.TU.Chr3.366		*evm.TU.Chr6.347*	*hypothetical protein BSL78_29306*	*evm.TU.Chr4.124*	*putative CUB and sushi domain-containing protein 2*	*evm.TU.Chr9.480*	
evm.TU.Chr3.365		*evm.TU.Chr6.346*	*hypothetical protein BSL78_29308*	*evm.TU.Chr3.527*		*evm.TU.Chr6.737*	*hypothetical protein BSL78_19792*
evm.TU.Chr3.364		*evm.TU.Chr6.345*	*putative GA-binding protein alpha chain*	*evm.TU.Chr21.93*	*hypothetical protein BSL78_14177*	*evm.TU.Chr5.480*	*putative cat eye syndrome critical region protein 5-like*
evm.TU.Chr23.322	*putative MAGUK p55 subfamily member 7 isoform X4*	*evm.TU.Chr5.649*	*putative heparan sulfate glucosamine 3-O-sulfotransferase 1-like*	*evm.TU.Chr2.750*		*evm.TU.Chr3.594*	*putative WSC domain-containing protein 2 isoform X1*
evm.TU.Chr20.134	*putative C-mannosyltransferase DPY19L1 isoform X2*	*evm.TU.Chr5.648*	*putative kinesin-like protein klp-3*	*evm.TU.Chr19.633*	*hypothetical protein BSL78_29032*	*evm.TU.Chr3.593*	*hypothetical protein BSL78_22192*
evm.TU.Chr2.1061	*putative nesprin-1-like*	*evm.TU.Chr4.1046*	*putative potassium voltage-gated channel subfamily KQT member 4 isoform X1*	*evm.TU.Chr19.632*	*putative radixin, partial*	*evm.TU.Chr23.218*	*Klf7*
evm.TU.Chr2.1060	*putative nesprin-1-like*	*evm.TU.Chr23.108*	*putative beclin-1*	*evm.TU.Chr18.314*	*hypothetical protein BSL78_07747, partial*	*evm.TU.Chr21.56*	*putative U2 snRNP-associated SURP motif-containing protein-like isoform X3*
evm.TU.Chr18.552		*evm.TU.Chr22.345*	*putative cadherin-23*	*evm.TU.Chr16.240*		*evm.TU.Chr21.55*	*hypothetical protein BSL78_26807*
evm.TU.Chr16.111	*putative RNA polymerase II elongation factor Ell*	*evm.TU.Chr20.477*	*putative alphaP integrin isoform X1, partial*	*evm.TU.Chr15.765*		*evm.TU.Chr2.1084*	*putative mitogen-activated protein kinase-binding protein 1*

evm.TU.Chr16.110	*hypothetical protein BSL78_22071*	*evm.TU.Chr2.1075*		*evm.TU.Chr15.764*	*hypothetical protein BSL78_02677, partial*	*evm.TU.Chr19.476*	*putative aminopeptidase N, partial*
evm.TU.Chr15.765		*evm.TU.Chr19.664*	*putative RING finger protein 17*	*evm.TU.Chr13.564*	*putative zinc finger matrin-type protein 2-like*	*evm.TU.Chr18.593*	*putative small subunit processome component 20-like, partial*
evm.TU.Chr15.764	*hypothetical protein BSL78_02677, partial*	*evm.TU.Chr16.724*	*putative tetraspanin-3*	*evm.TU.Chr13.563*	*putative Ras-related protein Rab-33 isoform X1*	*evm.TU.Chr16.305*	
evm.TU.Chr11.235	*putative required for meiotic nuclear division protein 1-like*	*evm.TU.Chr16.723*		*evm.TU.Chr1.590*	*hypothetical protein BSL78_04038*	*evm.TU.Chr15.765*	
evm.TU.Chr11.234	*putative serine/threonine-protein phosphatase*	*evm.TU.Chr15.765*		*evm.TU.Chr1.589*	*putative CCR4-NOT transcription complex subunit 11 isoform X2*	*evm.TU.Chr15.764*	*hypothetical protein BSL78_02677, partial*
evm.TU.Chr10.822	*putative riboflavin kinase*	*evm.TU.Chr15.764*	*hypothetical protein BSL78_02677, partial*			*evm.TU.Chr14.250*	
evm.TU.Chr10.821	*hypothetical protein BSL78_10663*	*evm.TU.Chr13.334*				*evm.TU.Chr13.548*	*putative E3 ubiquitin-protein ligase MYCBP2*
						evm.TU.Chr12.553	*putative 5′-3′ exoribonuclease 1-like*
						evm.TU.Chr1.368	*putative NLR family CARD domain-containing protein 4-like*

The blank areas represent proteins that had not been annotated previously and the bold font in-dicates genes that have been previously annotated as being associated with growth.

Table S2. Candidate genes associated with the papilla number traits in the G1 vs. G2 and G2 vs. G3 populations, respectively.

G1 vs. G2 population		G2 vs. G3 population	
**Gene ID**	**Gene Name**	**Gene ID**	**Gene Name**
evm.TU.Chr9.1058_evm.TU.Chr9.1059	*hypothetical protein BSL78_12224*	*evm.TU.Chr9.965*	*ATP-binding cassette transporter subfamily A*
evm.TU.Chr8.978		*evm.TU.Chr9.1040*	
evm.TU.Chr8.977	*hypothetical protein BSL78_11889*	*evm.TU.Chr9.1039*	*hypothetical protein BSL78_04387*
evm.TU.Chr7.928		*evm.TU.Chr8.1020*	
evm.TU.Chr7.927		*evm.TU.Chr8.1019*	
evm.TU.Chr6.819	*hypothetical protein BSL78_02281, partial*	*evm.TU.Chr7.938*	*putative PDZ domain-containing protein GIPC1-like*
evm.TU.Chr5.1105	*hypothetical protein BSL78_23414*	*evm.TU.Chr7.928*	

evm.TU.Chr4.1347		*evm.TU.Chr7.927*	
evm.TU.Chr4.1345	*putative peroxisomal acyl-coenzyme A oxidase 1 isoform X3*	*evm.TU.Chr5.1115*	*putative cellular nucleic acid-binding protein-like isoform X2*
evm.TU.Chr4.1344	*putative bystin-like*	*evm.TU.Chr5.1114*	*putative DNA/RNA-binding protein KIN17, partial*
evm.TU.Chr4.1338	*putative regulating synaptic membrane exocytosis protein 2 isoform X4*	*evm.TU.Chr3.751*	*tyrosine-protein kinase SRK2-like isoform X1*
evm.TU.Chr22.569	*hypothetical protein BSL78_05806*	*evm.TU.Chr3.750*	
evm.TU.Chr22.568		*evm.TU.Chr23.502*	*putative alanine--tRNA ligase, cytoplasmic*
evm.TU.Chr22.567	*putative S-crystallin SL11-like*	*evm.TU.Chr23.500*	*hypothetical protein BSL78_09278*
evm.TU.Chr22.555	*putative carboxypeptidase E-like*	*evm.TU.Chr22.620*	*hypothetical protein BSL78_12517*
evm.TU.Chr21.719	*diacylglycerol kinase delta-like isoform X2*	*evm.TU.Chr22.619*	
evm.TU.Chr20.608	*putative sterol O-acyltransferase 1*	*evm.TU.Chr22.618*	*hypothetical protein BSL78_12516, partial*
evm.TU.Chr20.607	*putative secernin-2*	*evm.TU.Chr22.617*	*cGMP-dependent protein kinase 1-like isoform X1*
evm.TU.Chr20.600	*putative testis-expressed sequence 10 protein-like*	*evm.TU.Chr22.599*	*histone H2A.V*
evm.TU.Chr2.1385	*putative alpha-actinin, sarcomeric-like*	*evm.TU.Chr22.555*	*putative carboxypeptidase E-like*
evm.TU.Chr19.694		*evm.TU.Chr21.719*	*diacylglycerol kinase delta-like isoform X2*
evm.TU.Chr19.693	*putative 28S ribosomal protein S9, mitochondrial isoform X1*	*evm.TU.Chr20.608*	*putative sterol O-acyltransferase 1*
evm.TU.Chr18.640	*putative caspase-6*	*evm.TU.Chr20.607*	*putative secernin-2*
evm.TU.Chr17.754	*putative coiled-coil domain-containing protein*	*evm.TU.Chr20.600*	*putative testis-expressed sequence 10 protein-like*
evm.TU.Chr17.751	*putative condensin complex subunit 3-like*	*evm.TU.Chr2.1385*	*putative alpha-actinin, sarcomeric-like*
evm.TU.Chr17.697	*putative small integral membrane protein 19*	*evm.TU.Chr19.688*	*trypsin-like serine protease*
evm.TU.Chr16.871	*putative solute carrier family 35 member G1-like*	*evm.TU.Chr18.560*	*hypothetical protein BSL78_19868*
evm.TU.Chr16.849	*putative ADAMTS-like protein 5*	*evm.TU.Chr18.559*	*putative organic cation transporter protein*
evm.TU.Chr15.772	*hypothetical protein BSL78_03033*	*evm.TU.Chr18.458*	
evm.TU.Chr13.784	*complement component C3*	*evm.TU.Chr17.527*	*hypothetical protein BSL78_01912*
evm.TU.Chr13.779	*hypothetical protein BSL78_16381, partial*	*evm.TU.Chr17.395*	*putative non-structural maintenance of chromosomes element 4-like A-like*
evm.TU.Chr13.778	*putative sushi, von Willebrand factor*	*evm.TU.Chr16.861*	*hypothetical protein BSL78_15732, partial*
evm.TU.Chr11.897	*hypothetical protein BSL78_02477*	*evm.TU.Chr16.860*	*hypothetical protein BSL78_15733*
evm.TU.Chr10.855	*putative carbohydrate sulfotransferase 11*	*evm.TU.Chr16.859*	*hypothetical protein BSL78_15734*
evm.TU.Chr10.1		*evm.TU.Chr14.570*	*vesicular glutamate transporter 1-like isoform X2*
evm.TU.Chr1.1191	*putative UDP-D-xylose:L-fucose alpha-1,3-D-xylosyltransferase 3-like*	*evm.TU.Chr13.712*	*15-hydroxyprostaglandin dehydrogenase*
evm.TU.Chr1.1181	*hypothetical protein BSL78_17589*	*evm.TU.Chr13.711*	*hypothetical protein BSL78_22469*
		evm.TU.Chr13.626	*putative motile sperm domain-containing protein 2*
		evm.TU.Chr12.596	*hypothetical protein BSL78_07737*
		evm.TU.Chr12.595	*hypothetical protein BSL78_07736*

	evm.TU.Chr11.981	
	evm.TU.Chr10.851	*mothers against decapentaplegic homolog 6-like*
	evm.TU.Chr1.827	*hypothetical protein BSL78_24560*
	evm.TU.Chr1.1168	*hypothetical protein BSL78_23407*
	evm.TU.Chr1.1167	*hypothetical protein BSL78_11565*

The blank areas represent proteins that had not been annotated previously and the bold font in-dicates genes that have been previously annotated as being associated with growth.
